# Protein Complex Discovery by Interaction Filtering from Protein Interaction Networks Using Mutual Rank Coexpression and Sequence Similarity

**DOI:** 10.1155/2015/165186

**Published:** 2015-01-27

**Authors:** Ali Kazemi-Pour, Bahram Goliaei, Hamid Pezeshk

**Affiliations:** ^1^Institute of Biochemistry and Biophysics, University of Tehran, Enghelab Avenue, P.O. Box 13145-1384, Tehran, Iran; ^2^Science College, University of Tehran, Tehran, Iran

## Abstract

The evaluation of the biological networks is considered the essential key to understanding the complex biological systems. Meanwhile, the graph clustering algorithms are mostly used in the protein-protein interaction (PPI) network analysis. The complexes introduced by the clustering algorithms include noise proteins. The error rate of the noise proteins in the PPI network researches is about 40–90%. However, only 30–40% of the existing interactions in the PPI databases depend on the specific biological function. It is essential to eliminate the noise proteins and the interactions from the complexes created via clustering methods. We have introduced new methods of weighting interactions in protein clusters and the splicing of noise interactions and proteins-based interactions on their weights. The coexpression and the sequence similarity of each pair of proteins are considered the edge weight of the proteins in the network. The results showed that the edge filtering based on the amount of coexpression acts similar to the node filtering via graph-based characteristics. Regarding the removal of the noise edges, the edge filtering has a significant advantage over the graph-based method. The edge filtering based on the amount of sequence similarity has the ability to remove the noise proteins and the noise interactions.

## 1. Introduction

Nowadays, the cellular biology researches have shifted from molecular to modular researches and the single gene or protein study is replaced with the gene or protein networks researches. The protein complexes are responsible for many vital processes within a cell. Proper identification of the protein complexes and modules is an essential step in the identification of the cell molecular and functional mechanisms. The analysis of the interactional network of the proteins (PPI), via clustering of proteins network, is one of the ways to understand the protein complexes. The graph clustering algorithms have several applications in the network analysis. The Markov Clustering algorithm (MCL), as one of the important clustering algorithms, was applied in some investigations to identify modules in the protein interactional networks. The MCL algorithm produces some noise clusters (clusters with no known complexes) or clusters with noise proteins that reduce the accurate predictions of the complexes. The quality of the protein interactional networks to some extent depends on the preparation method. For example, the results of the Y2H and the Mas are not completely overlapping. The error rate in the protein interactions obtained from the conventional methods, such as protein chips, the immunoprecipitation, and the two-hybrid system, is estimated to be about 40–90% [1]. Moreover, just 30–40% of the existing interactions in databases are related to the specific biological function [2]. In other words, most of the existing interactions lack a specific performance.

Protein data networks are static, whereas the functional module expresses a dynamic concept. Thus, the composition and the kind of proteins in a protein complex depend on the cell survival stage, the cell interaction, and the environment condition [3]. In other words, the interactions of the pair proteins in the interaction networks are not permanent. Despite this issue, these kinds of interactions have been applied in the PPI networks during the network clustering. Therefore, several interactions are stationed in the implemented clusters as noise. In this case, removing and modifying the clusters seem necessary.

Noise interactions could be eliminated from protein clusters by weighting interactions according to their differentiated weight. Various methods have been used to weight the interactions. Brun et al. used the graph characteristics for weighting the graph edges [4]. Lubovac et al. used the similarity scale of the GO between each pair of proteins for weighting the interactions [5]. Kritikos et al. weighted each interaction according to the way it was obtained [3]. Researchers showed that there is a significant relationship between the sequence similarity rate of each protein-pair and its rate of functional similarity in the protein-pairs of the protein interaction network [6, 7]. Joshi and Xu showed that, in protein-pairs with sequence identity equal to 30% or less, the possibility of observing the similar GO terms decreases [8]. Laboratory study results have shown that the amino acid sequence data of the proteins have sufficient capacity to predict the protein interactions [9–14]. In several studies, two criteria, identity and similarity, were used to study the protein sequences. Although the high identity rate between the two sequences represents their function similarity, the conclusion of the proteins' function similarity based on the identity rate of the sequence is usually inconclusive and baseless since the two sequence identities are usually very low [8]. In this research, we have introduced two new methods for weighting interactions in protein clusters to edit interactions and noise proteins.

## 2. Methods

### 2.1. Database and Clustering Method

The protein interaction network of Human Protein Reference Database (HPRD) [15] was used as the protein-protein interaction database. The Markov Clustering algorithm [16] was used to cluster the protein interaction network “HPRD” and to identify the protein clusters. Taking into consideration that Gavin et al. showed that the number of proteins in each module is between 5 and 10 [17], we have compared the clusters generated by the default values (inflation: 1.6, 1.8, 2, and 2.2) with the known protein complexes of the HPRD database to obtain the suitable influence parameters for the MCL algorithm. Given the number of clusters containing 5 to 10 proteins generated by the default inflation parameters (1.8, 2, and 2.2), no significant differences were observed between these clusters (of 5 to 10 proteins) in terms of the mentioned parameters. So, the value of 1.8 for the inflation parameter was considered.

### 2.2. Filtering Method

#### 2.2.1. Node Filtering

The selected clusters were compared with each other, using the two node filtering and edge filtering methods. For the node filtering method, the MCL-CA algorithm [18] was used. The MCL-CA algorithm identified the core and the attachment proteins in protein complexes based on the graph based characteristics of the protein network. In each cluster, the proteins, which have a higher edge degree than the average degree of the cluster edges, were selected as core proteins. Therefore, in the node filtering method, the proteins, in which their edge degree is less than the average degree of their cluster, were removed.

#### 2.2.2. Edge Filtering

*(1) Filtering Based on Coexpression*. The coexpression level of each pair of genes is considered the edge weight of the corresponding proteins with two genes in the PPI network. Pearson's correlation coefficient (PCC) is usually used to represent the coexpression of genes. Another indicator, which is used to present the coexpression of the two genes, is the rank index of the two genes towards each other. Since the rank of gene “A” to gene “C” (A → C) is not necessarily equal to the rank C → A gene, and due to the weakness of the Pearson correlation coefficient (PCC) in identifying and predicting the gene performance, as well as the GO interpretation [19], the geometric mean of the rank of the two coexpressed genes in regard to each other—“Mutual Rank” (MR)—was used to display the amount of the coexpression level of both genes (Table 1).

When the numerical amount of the MR is small, it indicates the strength of the coexpression of the two genes [20]. For each interacting protein-pair from HPRD, the MR was calculated, using the COXPRESdb database [20].

In the edge filtering method, based on coexpression, removing of the edge was performed based on the filtering of the edge containing the weak MR. The first neighbouring protein in the PPI network was identified and added to the proteins of each cluster to identify the threshold level of removing the edges.

Then, the characteristics, such as the average node degree (average node degree = (*E*/*N*) × 2), the edge density (edge density = (*E*/(*N* × *N* − 1)) × 2), (the number of edges: *E*, and the number of nodes: *N*), the average coexpression, and the functional score—in the clusters before and after the addition of neighbouring proteins—were compared with one another. The functional score was calculated by the “GraphWeb” web server, using the g:Profiler data [21, 22]. Once a cluster has been identified by the MCL algorithm, the GraphWeb automatically assesses its biological importance through the known properties of its members, using the g:Profiler software. Functional profiling of the cluster involves the statistically enriched annotations of biological processes, the cellular component, molecular functions, and related pathways.

The data used in the g:Profiler is derived from the Gene Ontology [23] and the pathways from the Kyoto Encyclopedia of Genes and Genomes (KEGG) [24] and Reactome [25] databases. The g:Profiler uses cumulative hypergeometric *P* values to identify the most significant terms corresponding to the input set of the genes, and it applies Fisher's test to evaluate the enrichments of all the biological annotations in the cluster. Once all the enrichments for the cluster are known, the “GraphWeb” computes an annotation score that sums the total significance relative to the cluster size *n*. The score is calculated by summing the logarithms of all the significant *P* values.

*(2) Filtering Based on Sequence Similarity Rate*. In each cluster, the protein-pairs with interactions were aligned together, and the similarity rate of each pair of proteins was considered the edge weight between the two proteins in the network. Then, the edges weighting less than 30 were excluded in each cluster. The BALIGN software, in the form of a Matlab program, was used to calculate the sequence similarity rate of protein-pairs with interactions [26]. BALIGN can be used to compute the pairwise alignments for the given lists of sequences. It can perform with both global [27] and local alignments [28] with affine gap penalties; and it can produce the Bit-score, the conservation score, and the percent identity matrices. We have calculated the similarity score for a pair of proteins, using the local alignment, and we will use the BLOSUM62 score matrix.

There is a direct relationship between similarity and Bit-scores; that is, the greater the similarity between the two proteins, the higher the Bit-score; also the function similarity between the two proteins generally increases as their sequence similarity increases over a broad range of Bit-scores [7, 29]. We applied a Bit-Score threshold of 30 for the edge filtering and, then, the edges weighing less than 30 were excluded in each cluster.

Properties, such as the number of nodes (proteins), the number of edges (interaction), and the functional score, have been measured before and after filtering, using the above two methods in each cluster. Then, the proteins were compared with the protein complexes and dense modules of the CORUM database [30] before and after filtering of each cluster; then, the numbers of the shared proteins with the known protein complex were calculated. In each cluster, properties, such as the number of nodes, the number of edges, and the functional score, were compared by the paired *t*-test procedure (Formula (1)) before and after filtering:

(1) $$\begin{array}{c} t=\frac{\sum d}{\sqrt{\left(n\left(\sum d^{2}\right)-{\left(\sum d\right)}^{2}\right)/\left(n-1\right)}}. \end{array}$$

Formula (1) is the paired *t*-test formula, where ∑*d* is the sum of the differences (i.e., the sum of *d*) and ∑*d* ^2^ is the sum of the squared differences.

To determine whether the protein is properly removed from the cluster by the filtering method is a binary classification problem.  Table 2 lists five measurements that are applied widely on evaluating binary classification problems. The accuracy is the most commonly used measurement.

**Figure 1:**
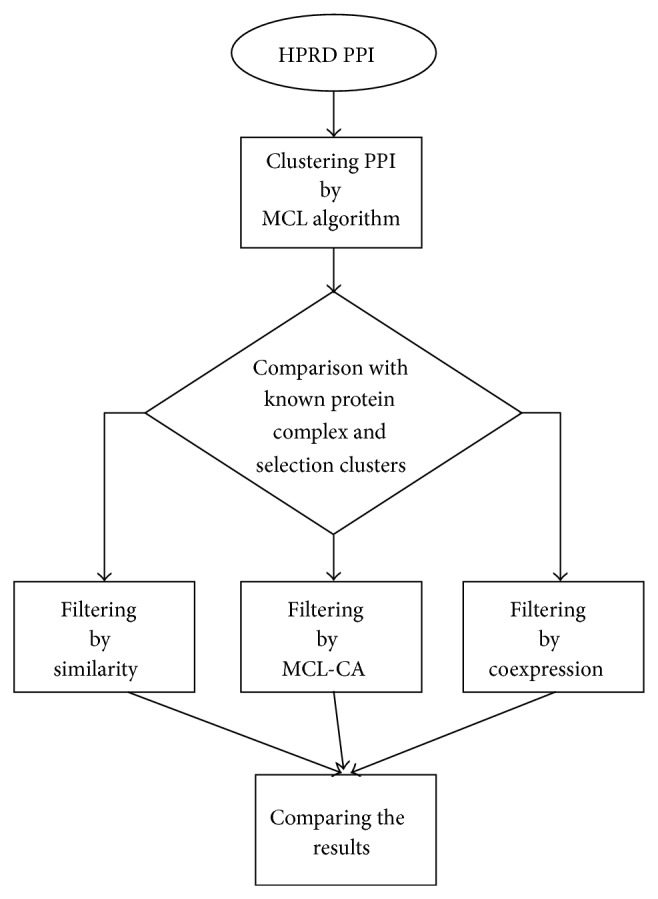
A flowchart of the steps of materials and methods.

Statistical analysis was performed, using the SPSS software. The steps of the materials and the methods are summarized in Figure 1.

## 3. Results and Discussion

From 1966 clusters produced by the clustering network (HPRD), 565 clusters had 5 to 35 proteins. While these 565 clusters were compared with the known protein complexes clusters of the HPRD database, 235 clusters with protein complexes were selected and analyzed.

Comparing these clusters before and after the addition of neighbouring nodes indicated that, by adding the neighbouring nodes, the mean value of the MR increased between an average of 800 and 1600 units per cluster. On the contrary, the amount of the functional score decreased between 16 and 40 points. This means that the added edges to each cluster had a high MR value.

**Figure 2:**
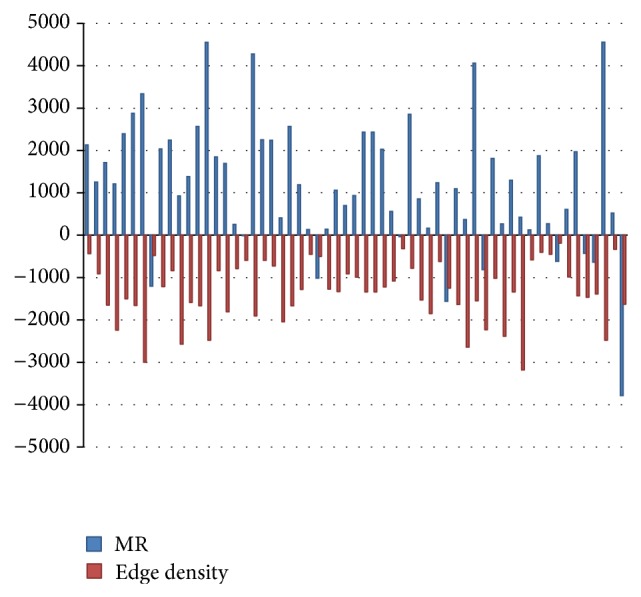
The negative correlation between the MR values and the edge density in protein clusters.

**Figure 3:**
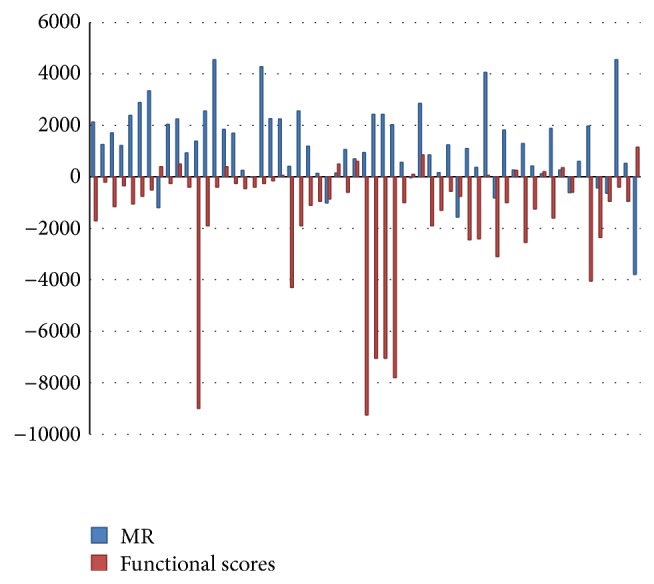
The negative correlation between the MR values with the functional score in protein clusters.

According to the negative correlation between the MR value of the edge density and functional score (Figures 2 and 3), the value of MR = 1000 was considered as the threshold level of the weak coexpression between the two genes. Therefore, the edges of each protein-pair in a cluster with the value of coexpression greater than MR = 1000 were removed. This was approved by Obayashi and Kinoshita [20] in a research that considered the coexpression with MR > 1000 as weak and unreliable.

**Figure 4:**
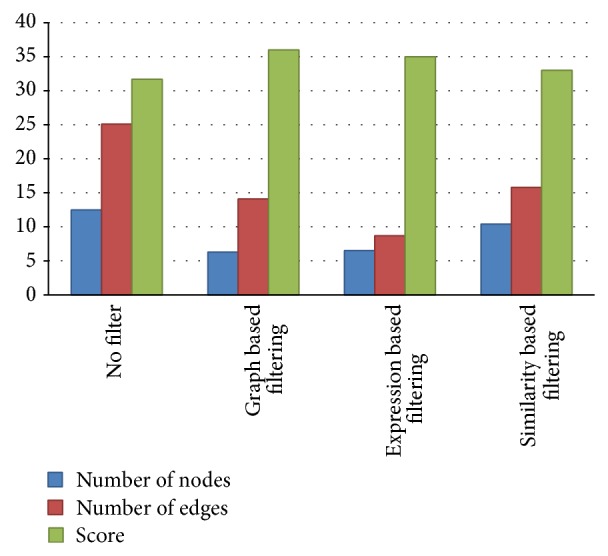
The effect of filtering methods on reducing the number of nodes and edges and changes in the functional score.

The statistical analysis of the node filtering method (based on the graph characteristics) and the edge filtering method (based on the gene expression) showed that both filtering methods significantly reduced the number of nodes, in terms of “the number of nodes per cluster” (Figure 4). The *t*-test results showed that there was a reduction of 4 to 8 nodes in 95 percent of the clusters, but there was no significant difference between the two approaches in the number of filtered nodes. In other words, the results of the filtering technique with both node and edge removing methods were identical in terms of the number of removed nodes.

Although the *t*-test results showed that there was no difference between these filtering methods in terms of the number of removed proteins, the comparison of the “sensitivity” in the two methods (Table 3) showed that the filtering based on the MR (sensitivity: 82%) had a higher capability of not removing the known protein complexes (TP) than that of the method based on the graph (sensitivity: 73%). On the other hand, the higher amount of the “specificity” based on the graph method (specificity: 70%) showed the capability of this method to remove other noncomplex proteins (TN).

The statistical analysis of the node filtering method (based on the graph characteristics) and the edge filtering method (based on the sequence similarity) showed that both filtering methods significantly reduced the number of nodes in terms of “the number of nodes per cluster” (Figure 4). However, the results showed that the number of eliminated nodes was greater in filtering by the node elimination method compared with filtering by the edge removal method, but in the method based on the similarity, the amount of sensitivity (94%) showed the higher capability of this method to not eliminate the known complex proteins. On the other hand, the capability of this method to eliminate the noncomplex proteins (TN) is low (specificity: 30%). The higher accuracy of both methods of the edge elimination, in comparison with the node elimination, shows the capacity of these methods in filtering the protein clusters and recognizing the protein complexes.

The statistical analysis of the effect of the three filtering methods on “the number of removed edges” indicates that the filtering methods significantly decreased the number of edges in clusters (Figure 4), but the edge reduction, using a coexpression factor, is more effective than that of the graph based approach; therefore, 95% of the clusters showed a decrease of 13 to 22 edges. However, in the node elimination method, the average number of edges removed was between 8 and 15, and the edge reduction, using the sequence similarity, showed a decrease of 6 to 12 edges in each cluster. In other words, there was a significant difference between the coexpression method and other filtering methods in terms of the reduction of the number of edges.

The statistical analysis of the three filtering methods on the “functional score” of clusters indicated that, in the filtering methods, there was no significant statistical difference between the filtered clusters and clusters with no filtering (Figure 4). This means that proteins and interactions that are eliminated from the primary clusters were without any significant performance associated with other cluster proteins. In other words, these proteins have no definite function in terms of GO, Reactome, and KEEG information or their performance is not similar to the remaining proteins.

**Figure 5:**
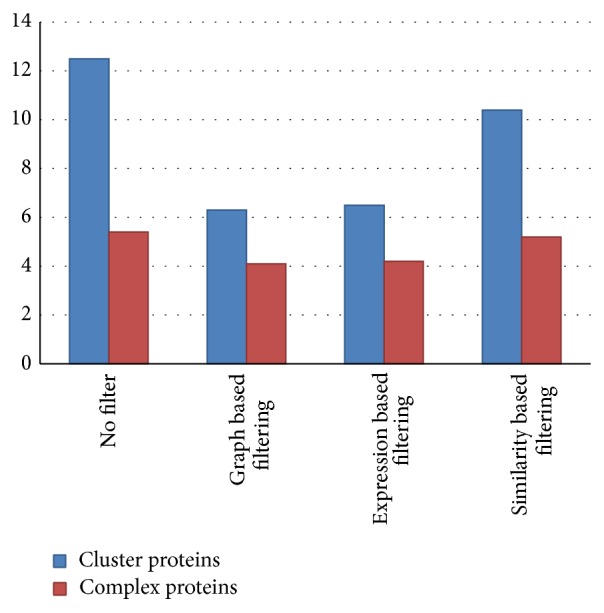
The effect of filtering methods on changes in the ratio of known proteins (complex proteins) to the total number of cluster proteins in each cluster.

The statistical comparison of the filtered clusters with the three methods of filtering, in terms of “the number of common proteins” of each cluster with the known proteins in protein complexes databases CORUM, showed that, in the methods of filtering, the ratio of the known proteins in the CORUM database to the protein number in each cluster has been improved in comparison with the nonfiltering methods, but there were no significant differences between the filtering methods. In other words, in the filtering methods, the removed proteins from the clusters were not included in the complex proteins (Figure 5).

**Figure 6:**
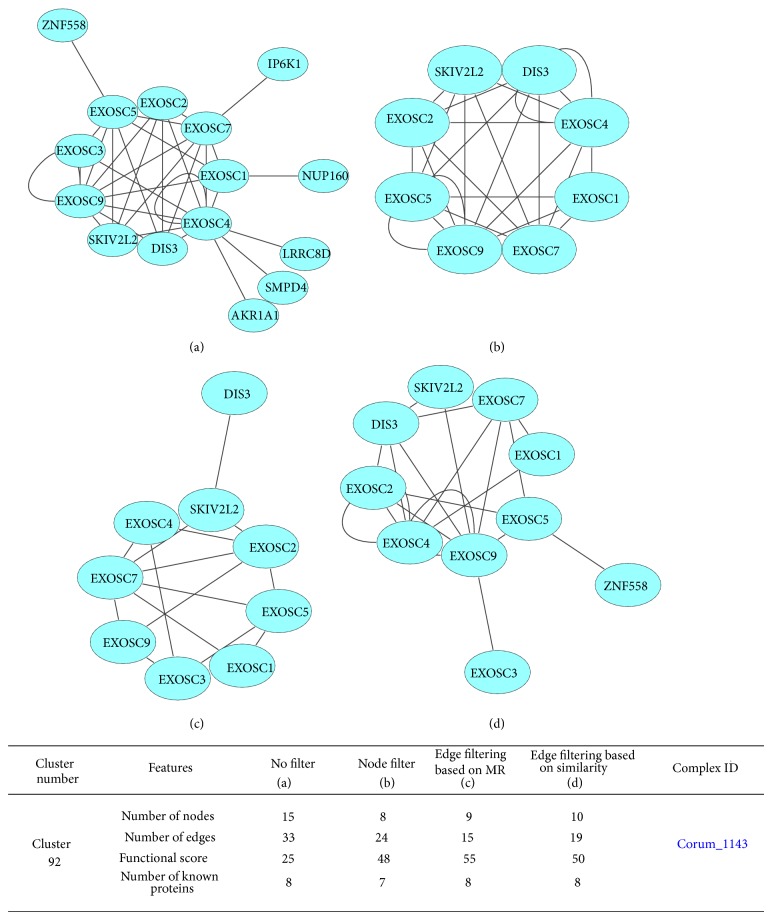
Images (a), (b), (c), and (d), respectively, indicate cluster 92, cluster 92 after filtering (using the node removal method), cluster 92 after the edge filtering method based on MR, and cluster 92 after the edge filtering method based on sequence similarity. Proteins of corum_788 complex belong to the REACT_20619.1 metabolic pathway, and they are involved in the metabolism of mRNA. Pictures were drawn by Sytoscape software [31].

For example, in cluster 92, the number of cluster edges decreased from 33 to 15 due to the edge filtering based on the MR and it decreased from 33 to 19 due to the edge filtering based on the sequence similarity. These decreases in the number of the edges caused the removal of 6 proteins in the MR base and 5 proteins in the sequence similarity base, while, in the node elimination method, 7 proteins, one of which (EXOSC3) belonging to the known proteins of the corum_788 complex, were removed (Figure 6).

## 4. Conclusion

The filtering method, using the coexpression index of the MR, has advantages compared to the node elimination method. The filtering technique based on the MR is more efficient than the graph based method in removing noise edges. This advantage becomes more important when we know that a large percentage (40% to 90%) of the existing interactions in the protein interaction network are noise. Although this method eliminated approximately 65% of the protein clusters' edge, no reduction in the number of known protein complexes and functional scores was observed.

The filtering method (using the sequence similarity index) has a biological basis, which is based on the principle that “the proteins amino acid sequence data indicate the structure, function, and interactions of the proteins.” Meanwhile, the node removal procedure is based solely on the topological properties of the PPI network graph.

On the other hand, the MCL-CA algorithm considers all the clusters, resulting from clustering, as potentially containing protein complexes, while regarding this default, filters cluster to identify the complexes. In each cluster, proteins with dense communications are considered as the core and proteins containing less communication are removed as noises. Based on the clusters from the network clustering in the CORUM database, it is concluded that any dense complex cannot necessarily be considered as a protein complex with a specific biological function. In each cluster, the presence of a core set as the default of the MCL_CA algorithm causes the algorithm to search all the clusters, created by the MCL, for core proteins.

However, using the filtering methods based on the MR, the clusters in which the amount of coexpression is at a high level (MR > 1000) and does not have any protein-pairs with MR < 1000 are eliminated as noise clusters at the start.

## Figures and Tables

**Table 1:** The PCC rank of gene A to gene C is 3, and that of gene C to gene A is 4. The MR between gene A and gene C is the geometric average of the ranks, as an alternative index for coexpression.

Genes A → C Rank 3			Genes C → A Rank 4		
Rank	PCC	Gene	Rank	PCC	Gene
1	1	Gene A	1	1	Gene C
2	0.96	Gene B	2	0.99	Gene D
3	0.95	Gene C	3	0.97	Gene B
4	0.92	Gene D	4	0.95	Gene A
5	0.87	Gene E	5	0.90	Gene G

$\text{MR}\left(\text{A},\text{C}\right)=\sqrt{\text{Rank}\left(\text{A}\to \text{C}\right)\times \text{Rank}\left(\text{C}\to \text{A}\right)}=\sqrt{3\times 4}=3.464102$.

**Table 2:** Evaluation measurements.

Measurement	Abbreviation	Equation
Sensitivity	Sens.	TP/(TP + FN)
Specificity	Spec.	TN/(TN + FP)
Precision	Prec.	TP/(TP + FP)
*F*-measure	Fm.	(2 Prec. Sens.)/(Prec. + Sens.)
Accuracy	Acc.	(TP + FN)/(TP + TN + FP + FN)

TP: true positives, FP: false positives, TN: true negatives, and FN: false negatives. TP: the number of known proteins (CORUM protein complex) that have not been properly removed by the filtering method. FN: the number of known proteins that have erroneously been deleted by the filtering method. FP: the number of unknown proteins that are not removed by the filtering method. TN: the number of unknown proteins that are removed by the filtering method.

**Table 3:** Accuracy values of the three filtering methods.

Filtering method	Sensitivity (%)	Specificity (%)	Precision (%)	*F*-measure (%)	Accuracy (%)
Based on MR	82%	62%	68%	74%	72%
Based on similarity	94%	30%	57%	70%	78%
Based on graph	73%	70%	71%	71%	67%
